# Distance Learning in Cameroon: Case Study of Private Nursery School Children's Experiences and Challenges Amidst COVID-19 Lockdown

**DOI:** 10.1007/s40841-022-00255-2

**Published:** 2022-06-01

**Authors:** Emela Achu Fenmachi, Rachel Ogene Awah Edah

**Affiliations:** 1 Division of Education, University of Waikato, Hamilton, New Zealand; 2 A Private Nursery School, Douala, Cameroon

**Keywords:** Distance learning, COVID-19, Children's experiences, Parental involvement, Socio-cultural concepts, Early childhood education

## Abstract

This article analyses data from a study that explored distance learning teaching and nursery school children's experiences in response to the COVID-19 pandemic in Douala, Cameroon. Following the spread of COVID-19 to Africa, the Cameroon Government placed emphasis on the impact of the pandemic on the socio-economic sector and actions to support this sector. There has been no reported research on the effects of the pandemic on the early childhood education sector and how children have experienced it. This article discusses distance learning techniques employed by teachers from a private nursery school and the views and feelings of young children whose teacher encouraged them to draw and tell stories about their experiences. Engaging in such conversations empowered and encouraged children to verbalise their COVID-19 Lockdown experiences. These conversations can help the teacher rethink and seek new ways to understand and guide children through challenging situations. In addition, the insights gained from the study can be helpful for policymakers concerned with maximising the capacity for schools and families to ensure continuous learning for all children in the event of a crisis.

## Introduction

While the Government's actions during the COVID-19 pandemic in Cameroon have been well documented on official websites (UNICEF, 2020), there has been no reported research on how nursery schools in Cameroon have responded to the pandemic or how nursery school children have experienced it. This article discusses distance learning opportunities offered by teachers in a private nursery school in Cameroon during the COVID-19 lockdown (March–August 2020). It then focuses on analysing children's drawings, conversations and stories about their experiences of COVID-19 in Cameroon, as told to their private nursery school teacher during and after the lockdown. Hearing children's voices enables an understanding of the effects of lockdown, what COVID-19 means to children, and how children can be nurtured and supported to develop resilience to cope with change. Listening to children allows them to express their ideas and opinions and participate in a democratic life where they have full rights and responsibilities (Pascal & Bertram, 2009). In addition, listening to children's "words give us the courage for the future and help us to find a new way to dialogue with the children and with ourselves" (Rinaldi, 2001, p. 1).

### Government's Response to COVID-19 in Cameroon

Following the COVID-19 outbreak in China, the disease rapidly spread to other regions of the world, including Cameroon in Sub-Saharan Africa. As of September 30, 2021, Cameroon had been seriously impacted by the disease, with over 92,303 confirmed cases and 1459 deaths (World Health Organisation, 2021) in a population of 26,857,715 (Worldometer, 2020). Cameroon recorded its first case of COVID-19 on March 6, 2020. The Government expeditiously acted by taking several measures to control the disease. Efforts were made to isolate initial cases and reduce spread through border closure, limiting movements between regions, putting in place curfews, testing and contact tracing of confirmed cases. These measures affected the education sector, as the Government announced the closure of all public and private training establishments at all levels of education, from nursery school to higher education, including vocational training centres and professional schools (Cameroon Tribune, 2020). The closure came into force on March 18, 2020 and remained in place until September, except for students sitting for examinations. Further measures were implemented on April 9, 2020. This included the mandatory wearing of face masks in public places, local production of medication, protective masks and hydro alcoholic gel, the establishment of specialised treatment centres, and scale-up in testing in all the regions. According to UNICEF (2020) reports, sanctions were placed on people found violating these measures.

During this period, the Government faced challenges such as: low capacity for testing; an inability to expand current intensive care units where expansion was needed; limited provision of ventilator support for patients in critical conditions; lack of effective contact tracing systems; and limited protective kits and supplies for medical personnel. There was no financial support for households and businesses. Furthermore, many people did not acknowledge the potential danger of the pandemic and refused to comply with confinement measures (Mbopi-Keou et al., 2020).

### Theoretical Framework

This study adopted a socio-cultural approach that explains how children know and understand the world through their activities and communication with others in a continuous learning process. The process of learning is not one-way but a reciprocal relationship in which both the adult and the child construct understanding and knowledge together (Smith, 2013). Bronfenbrenner's (1986) model is particularly relevant in showing how the home, schools, extended family, and society influence children's development. Bronfenbrenner's mesosystem comprises the interrelations among two or more settings such as the home, school, or a neighbourhood peer group in which a child actively participates (Bronfenbrenner, 1996). Thus, the child develops by interaction with the immediate environment. Therefore, parents, caregivers, or educators must build positive relationships and create a friendly and loving environment that significantly impacts the child's holistic development.

Bronfenbrenner's model fits well with Nsamenang's (2006) description of African conceptions of childhood, which explains the idea of learning and development through participation within the family and society. African conceptions of childhood define how African children learn and develop through self-generated efforts by participating actively in the family and the community (Nsamenang, 2006, 2008).

Therefore, as children engage and participate in daily activities, they develop their unique identities as well as skills, competency, agency, and autonomy (Colonna, 2012). From an African perspective, an individual needs to interact with others and assume social responsibility to attain full personhood (Nsamenang, 2006). The child's life is interconnected and interdependent with the family and community and is not a separate entity. Learning can effectively occur with participation and guidance offered by the family at home and the nursery school and lead to children's holistic development and a sense of self and personal identity.

## Research Context

Cameroon, located in West and Central Africa, operates two education systems, English and French, inherited from former colonial masters, Britain and France. The languages of instruction are English and French accordingly. The educational sector is both Government and privately owned, although most nursery schools are privately owned. Nursery school education is not compulsory, but it is recognised formally as Cameroon's first level of education. The age for admission into nursery education is 3–4 years. Nursery education lasts for 2 years, after which the child moves to the first level of primary school education (Ministry of Basic Education, 2018).

This article analyses data gathered with teachers and children in a private nursery school in Douala after it had closed its doors and resorted to distance learning following the Government's lockdown on March 18, 2020. Following the lockdown, the private nursery school mobilised resources to provide online learning opportunities for children and families and maintained communication.

The teachers in the private nursery school, in collaboration with families, follow a Deweyan philosophy that aims to co-construct educational and life experiences for children to become active members of society (Beckett, 2018). Teaching and learning are focused on a community-based approach, learning by hands-on activity, and the democratic parent forum where parents share ideas on children's learning and plan social activities that involve the school and families. Children's competencies are recognised by giving them awards and highlighting values of love and togetherness through yearly thanksgiving ceremonies.

The nursery school is an inclusive institution that includes children with special needs and employs teachers trained in special education studies. The age range of children in the nursery section is 3–6 years old, with a total number of 18 children and four female teachers.

### Sample

Ten children and four preschool teachers (including the headteacher) participated in the study. Data were gathered from early August 2020 to the end of October 2020.

The children who took part in the study were 5 and 6-year-olds, six of whom were boys and four of whom were girls. The school academic year for Cameroon begins in September, and so the 5-year-olds had been Nursery One (for 4 year-olds/younger) children during the lockdown and at the time of the data collection had now moved to Nursery Two for older children, and the 6-year-olds were former Nursery Two children who had since moved to primary one.

Among the four female nursery school teachers who took part in the study, two teachers were between the ages of 20–30 years old, one teacher between 31 and 40 years old, and one teacher was in the age group 41 years and above. All teachers were qualified nursery school teachers with teaching experience ranging from 5 to 20 years.

Initial discussions with teachers began in August 2020 through WhatsApp messaging, where the consent of teachers, parents, and their children were gained. Children's data was collected in September 2020, and follow-up questioning for additional information took place in October 2020. All private school teachers resumed duty from September 1, 2020, while the primary school section resumed on September 16 and the nursery school section on October 6, 2020. Thus, data collection for children was obtained in two ways: in the nursery school campus and through home visits by the headteacher. Initially, the headteacher interacted and gathered information from six former Nursery Two children now in year one on the school campus. Later, she undertook a home visit to collect data from four former Nursery One children who were then in Nursery Two.

## Methods

The study aimed to voice children's experiences and challenges during the lockdown. Listening and including children's voices in this study recognises them as active participants and not as passive subjects (James & Prout, 2015). Peters and Kelly (2015), in exploring multiple ways of seeing and knowing the child, noted that skills and flexibility are needed to understand children's perspectives and to hear their voice. McLeod et al. (2017) argue that active listening skills are required by the adult and an environment where children can develop their ideas and express themselves. Multimodal methods are well suited to finding out about children's perspectives because children can express themselves differently. Methods that have been found to be valuable for this purpose include observations, conversations, drawings, photographs, map making, guided tours, and listening posts (Clark, 2007; Clark & Moss, 2017; Pascal & Bertram, 2009).

According to Bland (2012), drawings provide rich data and strengthen the data obtained through discussion with participants. Children's drawings were used effectively to stimulate conversations and interpretation of ideas about their understandings of COVID-19 lockdown experiences in a New Zealand study by Kahuroa et al. (2021). Findings from this current study reveal children's knowledge of COVID-19, an understanding of how to keep safe and a sense of personal control in expressing their everyday experiences at home and around their communities.

This current study sought to find out:

1. In what ways did teachers in a private nursery school in Douala communicate with families and children during the COVID-19 lockdown?
2. What experiences and understandings did children convey to their teachers about the COVID-19 lockdown?
3. What opportunities and challenges were experienced by children during the lockdown?

This was a qualitative study with data gathered through:

- *Zoom interview* and WhatsApp messaging *with teachers.*
- *Children's drawings.*
- *Children's conversations with teachers about their drawings.*
- *Home visits by the headteacher to collect data from children who had not resumed study in the nursery school.*

In this study, the headteacher, who played a role as co-researcher, elicited stories and accounts of children's experiences through conversation and drawing techniques. Children were invited to draw a picture about their understanding of COVID-19 and their experiences during the lockdown and then explain the drawing to the teacher. The headteacher documented the explanations, and when all data was collected, the headteacher forwarded a transcript of children's conversations and associated drawings to the New Zealand author through WhatsApp messages. The discussions held by the headteacher with the children about their drawings enabled children to explain the meaning of their drawings and the headteacher to ask questions and seek clarification. In effect, the headteacher engaged in a participatory process with children, encouraging creative thinking and eliciting their responses to their drawings.

Information from teachers was obtained through Zoom interviews and WhatsApp messaging (Fenmachi, in press-b). Teachers were asked about how they communicated with families during this period, and the support offered to families and the distance learning opportunities provided to children during the lockdown.

### Ethical Considerations

Ethics must be considered in any research, especially that which directly involves human beings. Abed (2015) says ethics are moral principles guiding conduct and should not be considered only at the beginning of the research or fieldwork but should be born in mind throughout the research process. Ethical issues considered in this study involved access to participants, informed consent, considerations for involving children in the research, confidentiality and anonymity, participants' protection from harm, and participants' rights to withdraw. Approval to conduct research was obtained first from the University of Waikato, Division of Education Research Ethics Committee, and then from the Regional Delegation of Basic Education Littoral. To gain access to the parents, teachers, and children of the nursery schools, a letter was emailed to the school's principal explaining details of the study for their approval and consent. Discussions with parents and teachers took place through WhatsApp messaging, where I verbally explained to the research participants the purpose of the study and their rights before commencing any data collection. In this process, verbal consent was obtained from parents and teachers. The headteacher, in addition gained parents' and children's verbal approval before initiating any conversations with children. Information regarding families and the nursery school was kept confidential and reported using pseudonyms to keep participants anonymous and protect them from any potential risk. Participants voluntarily decided to participate in the study and had the right to discontinue at any stage of the data collection until they had approved the transcripts of their interviews.

### Data Analysis

Data were organised, transcribed, and coded using thematic analysis (Braun & Clarke, 2006; Creswell, 2018). Analysis was carried out in the following stages. First, open and closed-ended questions from WhatsApp messaging and then Zoom interviews were transcribed and coded. The codes were developed from the responses to the questions and words spoken by the teachers transcribed by the researcher and a transcript of children's conversations and associated drawings. The data were read repeatedly to understand and make meanings of the information.

## Findings

Data revealed the distance learning opportunities offered by teachers at the private nursery school. Children's stories revealed their knowledge about the symptoms of COVID-19 and COVID-19 prevention measures. Some child participants expressed emotions of fear and loneliness and described feeling uncomfortable with wearing facial masks and other social restrictions. However, they were excited about their online learning experiences and the opportunity of spending more time at home with their parents.

The following section reports on distance learning carried out by the private nursery school as a response to lockdown due to the COVID-19 pandemic. Following this, children's understanding of COVID-19 and experiences during the lockdown are discussed. Children in the study were asked to choose their pseudonyms to report the findings.

### Distance Learning as a Response to COVID-19 Pandemic

First, the interaction between the private nursery school and families during the COVID-19 lockdown is discussed. Due to the closure of schools, the private nursery school administration and teachers organised remote learning to engage with children and their families throughout the lockdown period. Teachers maintained contact with parents by phone calls and WhatsApp messages to check on children's wellbeing. The teachers in addition organised Zoom video conferencing sessions with teaching and learning activities and emailed worksheets to parents as homework. Zoom learning sessions were scheduled twice a week and lasted an hour each. Depending on the activity, Zoom sessions included singing, dancing, discussions, and practical hands-on activities like scissors, paper, and glue. In addition to Zoom sessions, teachers emailed worksheets for parents to print and assist children to complete for the rest of the week (Fenmachi, in press-a). Parents will later email completed worksheets to teachers for feedback and comments.

The shift from face-to-face to online learning was swift and posed challenges for teachers and families. Teachers acknowledged their limited digital skills in carrying out online learning. Nevertheless, the COVID-19 lockdown encouraged teachers to seek alternative ways of teaching and interacting with children and their families. Teachers reported that parents, despite challenges faced during this period especially in their businesses, coping with work and home schooling, expressed delight over the opportunities for ongoing learning provided by the school. In addition, though many children conveyed they embraced distance learning with excitement, they also missed face-to-face learning and the company of their friends and teachers. Their stories indicated that they were overwhelmed with current restrictions of wearing masks in public places and social distancing practices.

### Children's Stories and Experiences During the Lockdown

Children's experiences were categorised into what they knew about the virus, the changes that occurred due to the outbreak of the virus, how they felt about these changes, and their hopes for the future.

Children's description of the virus indicated that they understood a lot about the symptoms as well as prevention measures of the disease. Children expressed that COVID-19 caused people to be sick, have a cough, headache, fever, and runny nose, and cause death. In order to avoid the disease, people must stay at home, wear masks, avoid handshakes and hugs, wash hands and use sanitisers. Children's comments indicated that they embraced distance learning with curiosity and excitement, though they missed the practical experiences associated with face-to-face learning. Children also enjoyed exploring new technology and learning methods. Still, they sometimes felt challenged with the new obligatory requirement of wearing a mask whenever they stepped out of home and social distancing practices. They appeared to miss their former freedom of intimacy, freedom of movement and longed for the day they could return to school and play with their friends. Children's stories showed excitement about new learning opportunities, views, and ideas about COVID-19, expressions of fear and loneliness, challenges with social restriction measures. The themes are reported below with examples of children's individual stories.

### New Learning Opportunities

Children in the study reported with excitement the new opportunities for learning introduced by their teachers during the lockdown. When asked about what they missed and enjoyed during the lockdown period, Ann (a 5-year-old girl) responded that she stayed home and ate, slept, watched TV, and used her Tablet. She was "so happy" to study on Zoom with her friends and teacher. In addition, she listed the different routines and experiences during the lockdown "I feel good staying at home, but I miss my school. I miss writing and learning at school. In my home, I learnt how to wash plates and make up my bed." These are characterised as self-acquisition skills relating to African concepts of learning and development. Parents instil independence skills in their children by allowing them to participate in the day-to-day activities of the home, working together for the general cohesion of the family.

The private nursery school teachers organised Zoom learning sessions during this period and emailed worksheets to parents for continuous learning after Zoom sessions. The children were comfortable with distance learning although some mentioned how they had missed face-to-face learning at the nursery school. Carl (a 6 year-old boy) reported, "I have been studying on my computer and watching TV. I feel happy to come back to school because I can learn with my friends." Aria (a 6 year-old girl) added:

I talked on Zoom with my teacher. I have never talked on the computer with my teacher, so it makes me feel excited. I played at home too. I am happy to stay at home. I sleep longer. But I miss my friends. I want to go back to school soon. I want to see my friends and my teacher.

### Views and Ideas about COVID-19

**Fig. 1:**
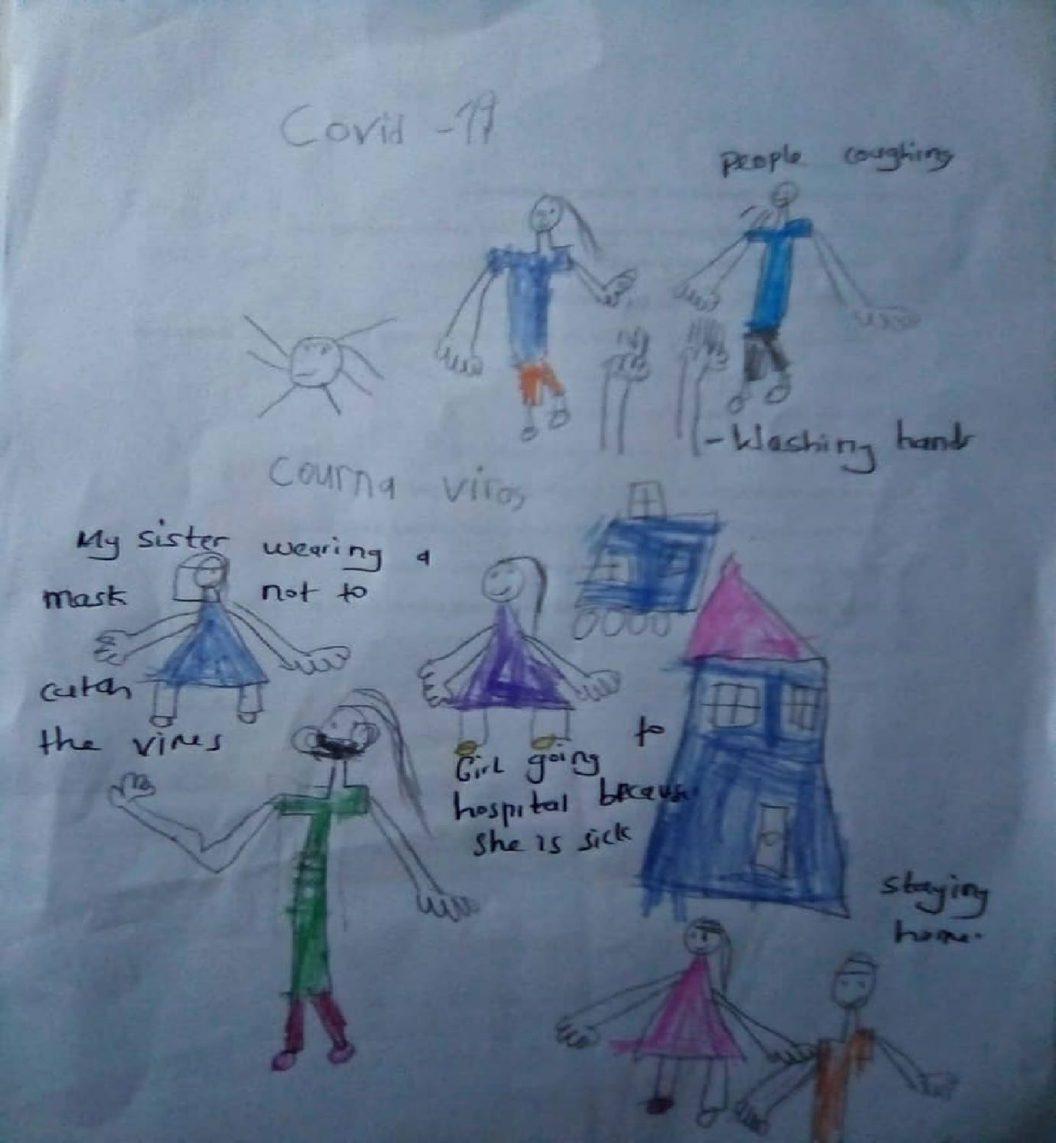
John’s drawing on COVID-19 prevention measures

**Fig. 2:**
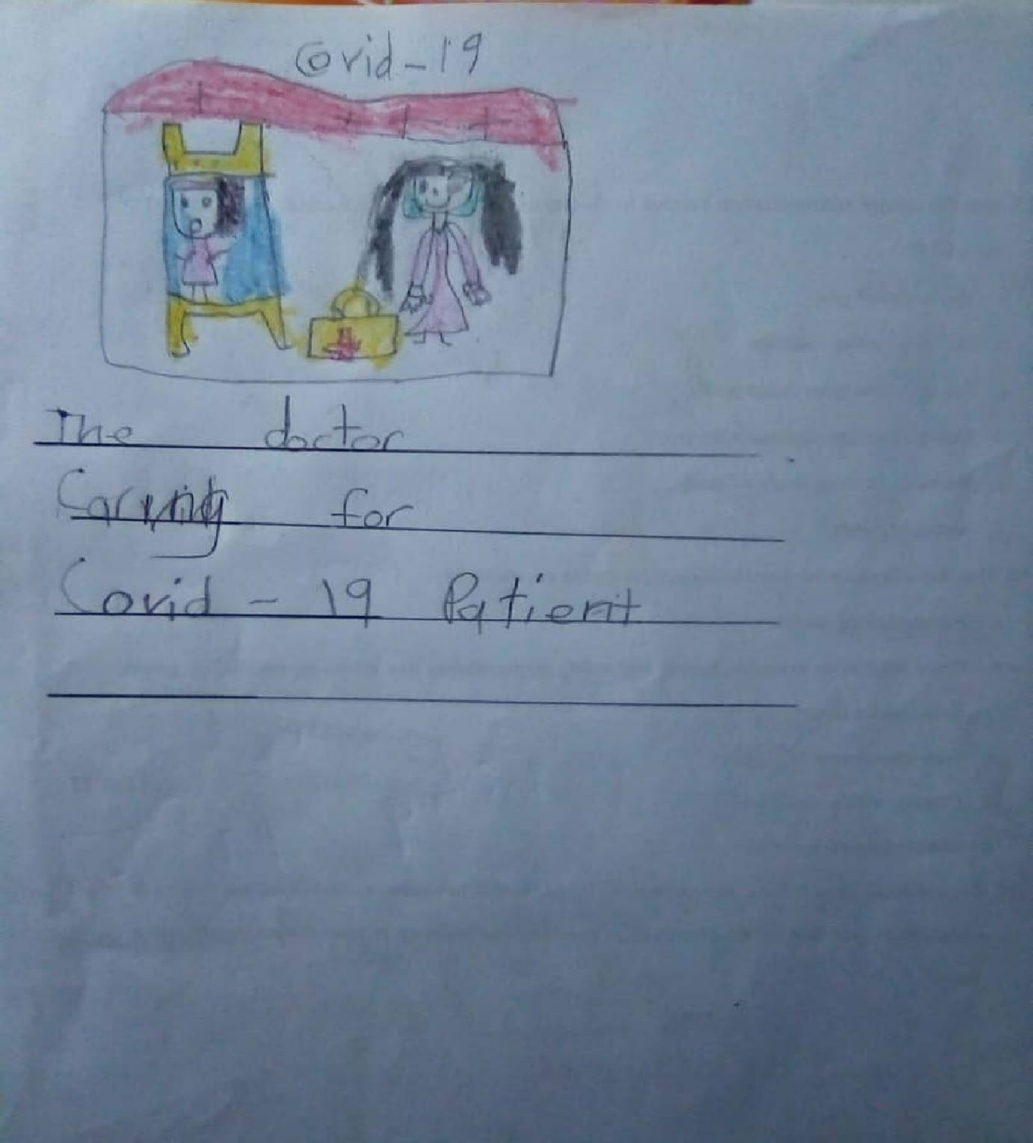
Levi’s drawing of a Doctor administering care to a COVID-19 patient

Children's stories conveyed knowledge and understanding about COVID-19, the symptoms associated with COVID-19 as well as COVID-19 prevention measures. Ann's (a 5-year-old girl) story showed awareness of COVID-19 symptoms as well as infection prevention. "COVID-19 means to cough, to have a headache, to have a fever, running nose. I cannot give my friends a handshake and kiss them because of COVID- 19." John (a 6-year-old boy) listed some measures on how to protect oneself and prevent the spread of the virus (Fig. 1). "Because of COVID-19, we must wear masks, must wash our hands, we don't greet, we use hand sanitiser." Levi (a 6-year-old boy) listed measures in which the virus can be prevented and expressed hopes in the doctors for helping COVID-19 patients (Fig. 2).

I think COVID-19 is a kind of germ that can make people very sick. I stayed one meter away from my sister even at home. I always washed my hands with soap for 20 seconds and wore my mask all the time. I think COVID-19 will stay, but doctors will treat sick people.

### Expressions of Fear and Loneliness

Some children expressed fear and loneliness during the lockdown period. Bibi (a 6-year-old girl) expressed emotions of fear and loneliness in her story as she states,

COVID-19 makes people die. I feel so scared because I must stay at home because I don't want to die. I played at home. I played upstairs, and I played downstairs too. I feel bored because I don't have a friend to play with.

**Fig. 3:**
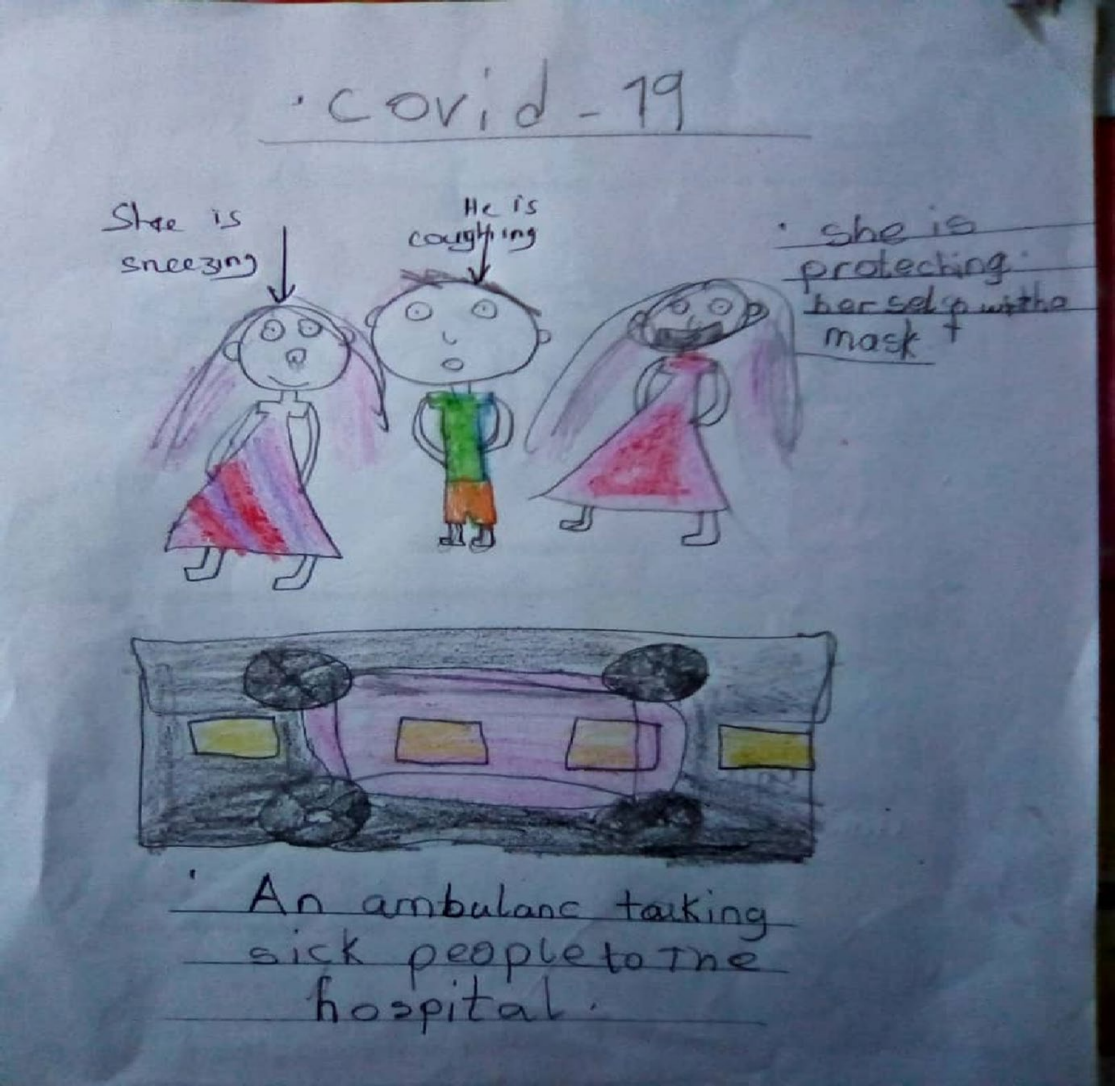
Bibi’s drawing of people sick from COVID-19

The expression of fear is further illustrated in Bibi's drawing (Fig. 3) as she draws people experiencing symptoms of COVID-19 and an ambulance taking patients to the hospital. She conveyed that even though she is happy to go back to school and meet her friends, there will be no freedom or intimacy as she must wear a mask to keep safe.

Leo (a 5-year-old boy) described what the virus might look like in his story. He expressed fear of contracting the virus, so staying indoors to be safe was the only remedy.

COVID-19 is a germ-like ball with spikes. I stayed at home playing and praying. I prayed to God to save my family and friends from the bad sickness. I played with my sister and my baby brother. We always stayed in my house because if we went out, we could meet the virus.

### Challenges with Social Restriction Measures

**Fig. 4:**
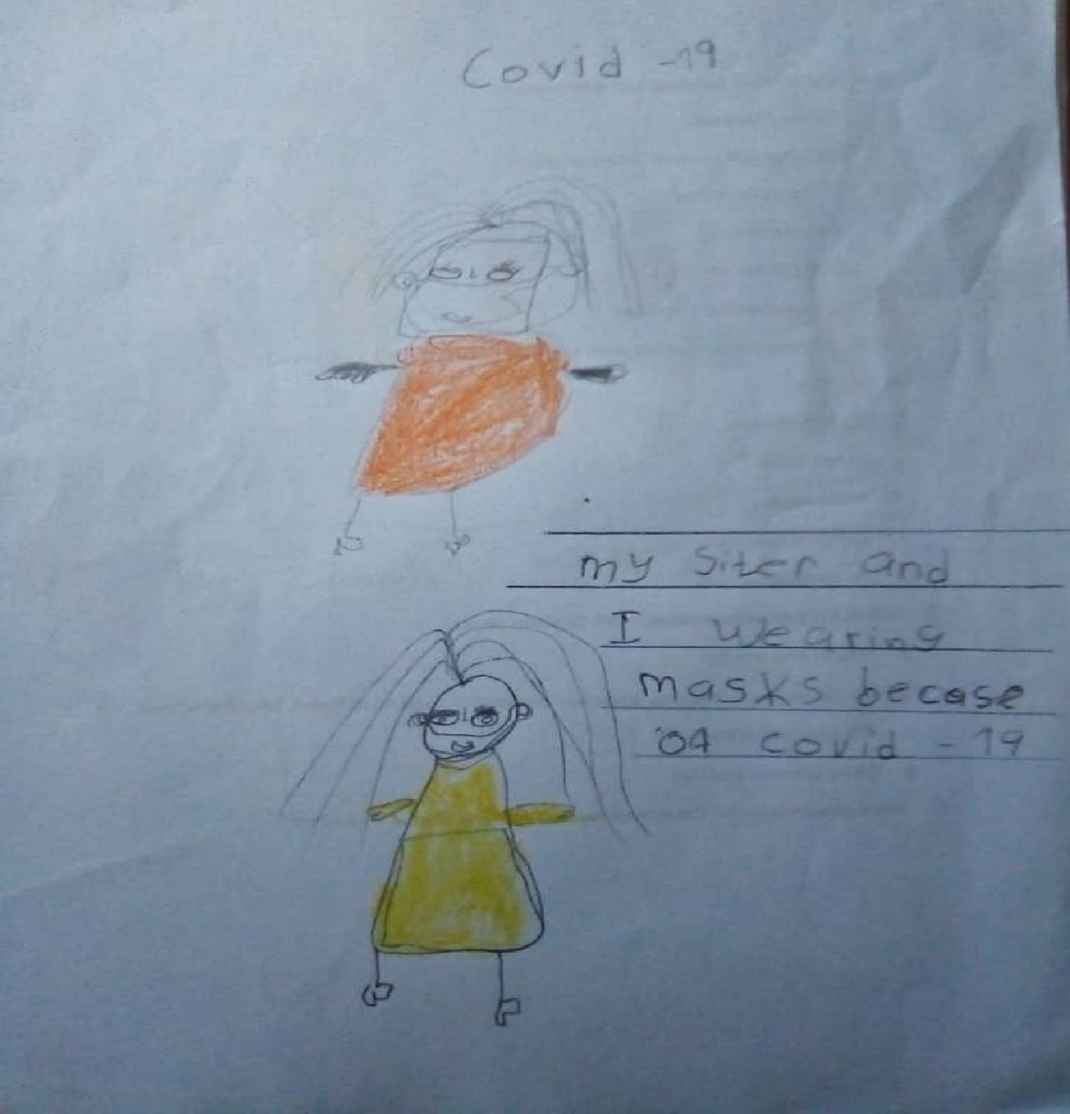
Brian’s drawing on the wearing of facial mask

Coupled with emotions of fear and loneliness were the challenges children face with the new social restriction measures, noted earlier and put in place by the Government. Ann (a 5-year-old girl) reported, "I cannot give my friends a handshake and kiss them because of COVID-19." Emma (a 6-year-old girl) said, "COVID-19 causes people to wear masks. If you get the virus, you will feel sick, you will cough a lot and have a runny nose." Similar to Emma's story, Brain (a 5-year-old boy) shares ways in which the virus can be prevented by wearing a facial mask and staying at home (Fig. 4). "COVID-19 means we should not go out without a mask, and we should stay home."

**Fig. 5:**
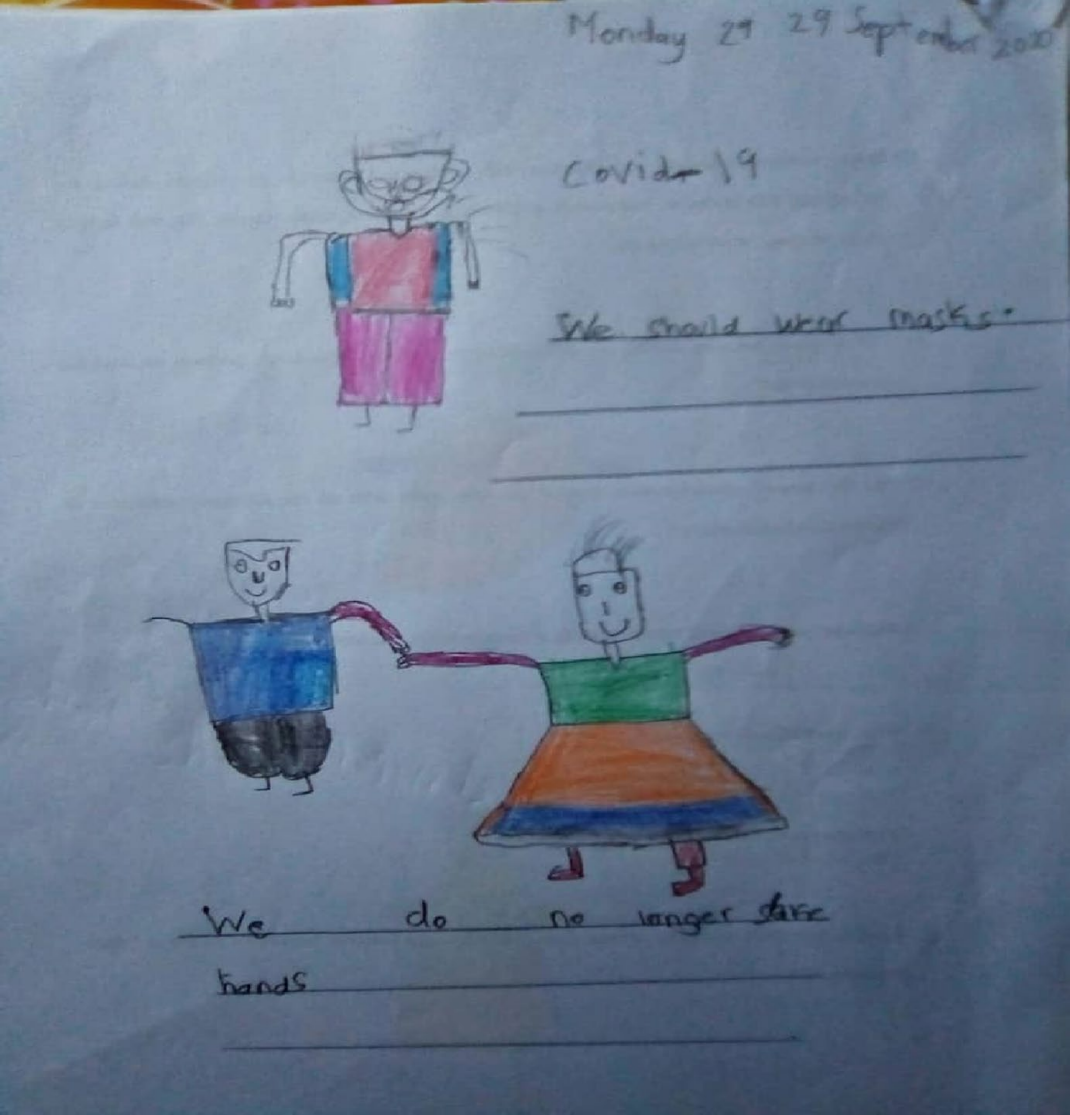
Jack’s drawing on COVID-19 prevention measures

Nevertheless, there was evidence that some of the children decided not to dwell on their past COVID-19 experiences but were hopeful for what the future might bring. Carl (a 6-year-old boy) recounts the idea of wearing a mask and shares his beliefs for the future. "COVID-19 is a virus that makes people sick. We have to wear masks to protect ourselves. I think everyone will be happy when COVID-19 ends." Although Jack (a 6-year-old boy) was a little sad over the new restrictions put in place as a result of COVID-19 (Fig. 5), he also hopes the virus will be defeated with the help of doctors. "We stopped going to public places and restaurants. I stopped visiting friends and travelling. I feel a little sad. I think COVID-19 will stop. The doctors will find a cure."

### Study Limitations

Limitations to note in this study centre around the method and data collection process. Information about children's experiences was not obtained directly by the researcher. The preschool headteacher collated children's stories and shared this with the researcher via Zoom and WhatsApp messaging. Thus, the presence of the preschool teacher may have influenced children's responses and parents' consent for children's participation. Another limitation is the sample size. The findings represent information from one private preschool only. Nevertheless, the study contributes valuable knowledge regarding preschool children's experiences during the lockdown period, as there is currently no research on how preschool school children in Cameroon have experienced the pandemic.

## Discussion and Conclusion

The article's main aim was to explore and voice children's stories and experiences during the lockdown. Engaging children through drawing and conversation techniques encouraged them to face their fears and express hopes for the future. Engaging in conversations about children's COVID-19 experiences also creates opportunities for caregivers, educators, and policymakers to rethink and seek new ways to understand and guide children through future challenging situations. The study revealed signs of distress in children as they expressed emotions of fear and loneliness in their stories, their inability to socialise with peers and engage in face-to-face learning, and other restrictions like wearing face masks. Nevertheless, the lockdown presented opportunities for children and parents to spend more time together, and children enjoyed exploring different learning techniques. Teachers in partnership with parents were able to create an online learning environment for children through Zoom sessions and emailing of worksheets. Parents ensured children were supervised throughout the teaching and learning process and forwarded completed worksheets to teachers for feedback.

One significant finding that emerged from children's stories was their ability to engage in learning at home (washing plates, making up their beds) in addition to what teachers had taught them. Parents encouraged their children to carry out some tasks at home to promote self-reliance and responsibility. Usually, tasks ranged from fetching water, clean-up after meals, washing, assisting in the kitchen and caring for younger siblings. Participating in the daily routines of the home is an essential aspect of child development and learning in the African context (Colonna, 2012; Nsamenang, 2008), which was not drawn on in teacher-led activities in this study. It is established in the literature that culturally responsive education enhances children's learning experiences and learning outcomes (Cooper et al., 2014; Fa'avae, 2017). Thus, it is highly recommended that early childhood education programs draw from indigenous knowledge and material resources within the local environment (Garcia et al., 2008).

The findings show the significant role of teachers as they actively engaged children and communicated with families during this period and the efforts made by parents in participating in children's learning. Children's ability to engage in face-to-face learning and interaction with teachers and peers were significantly altered due to COVID-19. Nevertheless, children in this study could still interact virtually with their families, extended family, friends, and teachers. Close connection and communication between the two microsystems (home and nursery school) positively influenced children's learning and development (Bronfenbrenner, 1996). In line with international perspectives (Mitchell et al., 2020; Park et al., 2020), teachers have played an important role by providing education and care for children of frontline workers as well as offering online learning opportunities for children during the lockdown. Collaboration between teachers and families enhances children's learning and forms strong connections between children, parents, and teachers (Fenmachi, 2020).

The private nursery school teachers had no initial training in using digital technology to carry out online learning with children. Although Zoom sessions involved activities such as singing, dancing, discussions, the activities were designed for the whole class setting and not according to each child's particular need. Considering children's developmental levels and finding the appropriate online learning tools to promote children's participation and learning (Kim, 2020) are some key points that teachers would have understood if previously trained to engage in online learning. Therefore, Government action concerning implementing ICT-related learning opportunities and courses that include the skills and knowledge needed for online teaching for in-service and pre-service teachers is essential.

Finally, the private nursery school's initiative to organise distance learning with children and families during the lockdown serves as an exemplary situation to other early learning institutions in Cameroon. This is especially true for Government-led institutions where limited or no opportunities were provided for communication and continuous learning experiences away from the nursery school to children and their families. Some of the barriers to organising distance learning in Government-led nursery schools was high pupil-teacher ratios and limited access to computers and the internet for some low-income families. The Government needs to consider recommendations by UNICEF (2019) to enforce quality learning services such as lower pupil-teacher ratios and an increase in investment related to resources for learning and teaching. The COVID-19 pandemic imposed hardship on many families and limited the opportunities for low-income families to provide extra educational resources for children. Government action that targets income-related support to especially low- income families and children's access to digital learning resources is necessary to ensure continuous learning for all children in the event of a crisis (Fenmachi, in press-a).

## Data Availability

Data generated during the current study are available from the corresponding author upon request.
